# Socioeconomic status and equity among patients with cardiogenic shock

**DOI:** 10.3389/fcvm.2025.1597225

**Published:** 2025-09-09

**Authors:** Marta Marcos-Mangas, Teresa López-Sobrino, Albert Ariza-Solé, Ferran Rueda-Sobella, Esther Sanz-Girgas, Jaime Aboal, Pablo Pastor, Irene Buera, Alessandro Sionis, Rut Andrea, Judit Rodríguez-López, Carlos Tomas, Jordi Bañeras, Isaac Llaó, José Carlos Sánchez-Salado, Cosme Garcia-Garcia

**Affiliations:** 1 Cardiology Department, Hospital Universitari Germans Trias I Pujol, Badalona, Spain; 2 Cardiology Department, Hospital Clínic de Barcelona Institut d’Investigacions Biomèdiques August Pi I Sunyer (IDIBAPS), Universitat de Barcelona, Barcelona, Spain; 3 Cardiology Department, Hospital Universitari Bellvitge, L’Hospitalet de Llobregat, Spain; 4 Cardiology Department, Hospital Universitari Joan XXIII, Tarragona, Spain; 5 Cardiology Department, Institut Investigació Sanitaria Pere Virgili, Tarragona, Spain; 6 Cardiology Department, Hospital Universitari Josep Trueta, Girona, Spain; 7 Cardiology Department, Hospital Arnau Vilanova, Lleida, Spain; 8 Cardiology Department, Hospital de la Vall d’ Hebrón, Barcelona, Spain; 9 Cardiology Department, Hospital Santa Creu I Sant Pau, II-B Sant Pau, Barcelona, Spain

**Keywords:** socioeconomic status, cardiogenic shock, intensive cardiac care units, mortality, intensive care management

## Abstract

**Background:**

We aimed to analyze the impact of socioeconomic status (SES) on management and in-hospital outcomes of patients with cardiogenic shock (CS).

**Methods:**

This was a prospective observational registry conducted (December 2018–November 2019) in Intensive Cardiac Care Units (ICCU) across 8 tertiary care centers. Consecutive patients aged ≥18 years with a primary diagnosis of cardiogenic shock were included. SES was defined using a numerical index that incorporates mean income levels, premature mortality, and avoidable hospitalizations observed within a specific health area. SES values were categorized into tertiles. In-hospital procedures, complications, length of stay, and in-hospital mortality were collected.

**Results:**

A total of 382 patients were included (mean age: 65.3 years). There were no differences in age, sex, or major comorbidities across SES groups. CS was more frequently due to acute coronary syndrome (ACS) in patients with low SES (66.9% vs. 58%, *p* = 0.022). No significant differences were observed regarding SCAI stage or other severity markers of CS across SES groups. Patients with low SES were more likely to receive pulmonary artery catheterization (*p* = 0.029) and mechanical circulatory support (*p* = 0.038). After adjusting for potential confounders, clinical management was similar regardless SES. Lower SES patients exhibited a higher incidence of bleeding (*p* = 0.018). There were no differences in length of stay or in-hospital mortality among SES groups.

**Conclusions:**

Beyond a higher rate of ACS-related CS, patients with low SES exhibited a clinical profile and shock severity comparable to other SES groups. Therapeutic management aligned with guideline recommendations even in patients with low SES.

## Introduction

Cardiogenic shock (CS) is associated with high morbidity and mortality rates and significant healthcare resource utilization (1, 2). One of the key recommendations in clinical practice guidelines is to transfer patients to centers with full availability of interventional cardiology and mechanical circulatory support (MCS), advocating for the organization of care through regional networks that integrate centers of varying complexity (3). Moreover, an association has been described between lower socioeconomic status (SES), differences in therapeutic management, and worse outcomes in patients with cardiovascular disease (4–7). However, data on the impact of SES in CS remain scarce. Most available information originates from administrative databases (8–11), which lack critical clinical variables. Furthermore, most published studies come from countries with healthcare systems significantly different from the free and universal Healthcare Spanish system. Therefore, the primary objective of this study was to assess the impact of SES on clinical management and in-hospital outcomes in a cohort of consecutive patients with CS admitted to Intensive Cardiac Care Units (ICCU).

## Methods

### Study population

The *Shock-CAT* registry is a prospective observational study performed between December 2018 and November 2019 in the ICCU of eight Spanish university hospitals (12). All participating centers had dedicated ICCU staffed by personnel with specialized training in the management of critically ill cardiac patients. Additionally, all centers had full access to interventional cardiology laboratories, and most of hospitals (75%) had on-site cardiac surgery and advanced MCS availability. The study included consecutive patients aged ≥18 years with a primary diagnosis of CS due to various etiologies.

CS was defined as a systolic blood pressure <90 mmHg (after adequate volume resuscitation) for at least 30 min or the need for vasopressor therapy to maintain a systolic blood pressure >90 mmHg, along with signs of hypoperfusion (altered mental status/confusion, peripheral coldness, oliguria, or lactate >2 mmol/L), in accordance with the classical definition of CS (13). Patients with postcardiotomy CS or those in whom CS was related to non-cardiac surgery were excluded. The underlying cause of CS was determined locally by investigators based on the primary admission diagnosis. CS management was performed according to current guideline recommendations (3) and at the discretion of the treating medical team.

### Data collection and definitions

Data were prospectively collected by local investigators using standardized case report forms. Collected variables included demographic data, baseline clinical characteristics, echocardiographic and angiographic findings, laboratory results, the need for invasive procedures during hospitalization, and the occurrence of in-hospital complications (bleeding, infections, arrhythmic and mechanical complications, severe brain damage, and in-hospital mortality). Advanced mechanical circulatory support (aMCS) was defined as the use of venoarterial ECMO, Levitronix Centrimag® or Impella® devices. MCS included both aMCS and the use of intraaortic balloon pump. Hospitalization events were assigned through a review of electronic medical records. The Cardshock score was calculated for each patient (14). CS severity was evaluated using the SCAI classification (15), with SCAI stages D and E categorized as profound shock. SES was defined using a numeric index developed by *the Agència de Qualitat i Avaluació Sanitàries de Catalunya* (AQuAS) (16). This index ranges from 0 to 100 and is collectively assigned to the entire population from a specific healthcare area (*Àrea Bàsica de Salut*). It incorporates factors such as average annual individual income, premature mortality, and avoidable hospitalizations within each healthcare area. Higher index values correspond to lower SES. For the purpose of this study SES values were categorized into tertiles (high SES: <39; medium SES 39–52, low SES >52). Despite its use has not been valitaded among patients with CS, this score has been associated with other healthcare indicators at an ecological level (17).

### Ethical considerations

Confidential patient information was protected in accordance with national legislation. This study was approved by the corresponding Institutional Ethics Committee. All study procedures were conducted in compliance with the ethical standards outlined in the Declaration of Helsinki. All patients provided written informed consent for the use of their clinical data for research purposes. In cases where patients were comatose or unable to provide consent, consent was obtained from their relatives.

### Statistical analysis

Results are presented as absolute numbers (*n*) and percentages (%). Categorical variables are expressed as frequencies and percentages, while continuous variables are reported as median and interquartile range (p25–p75). Comparisons of clinical characteristics, treatment variables, and prognostic outcomes across SES groups were performed using the *χ*^2^ test for categorical variables and analysis of variance (ANOVA) for continuous variables, including a test for linear trend.

The potential association between SES and in-hospital management was also assessed by multivariate analysis. Binary logistic regression models were performed considering main in-hospital procedures [MCS, angiography, percutaneous coronary intervention (PCI), therapeutic hypothermia, invasive mechanical ventilation, pulmonary artery catheter, respectively] as dependent variables, SES as a fixed independent variable and the rest of potential confounders (variables with an association *p* < 0.2 both with SES and in-hospital procedures) as independent variables. The association between SES and mortality was assessed by the same method. A two-tailed *p* value <0.05 was considered statistically significant. Statistical analyses were conducted using IBM SPSS Statistics 21 (Chicago, Illinois, USA).

## Results

A total of 382 patients were included, with a mean age of 65.3 years (SD 14). The majority of cases were male (287/382, 75.1%). The most frequent cause of CS was acute coronary syndrome (ACS) (232/382, 60.7%). Profound CS was observed in 139/382 patients (36.4%), and 133 cases (34.8%) experienced cardiac arrest during hospitalization.

**Figure 1:**
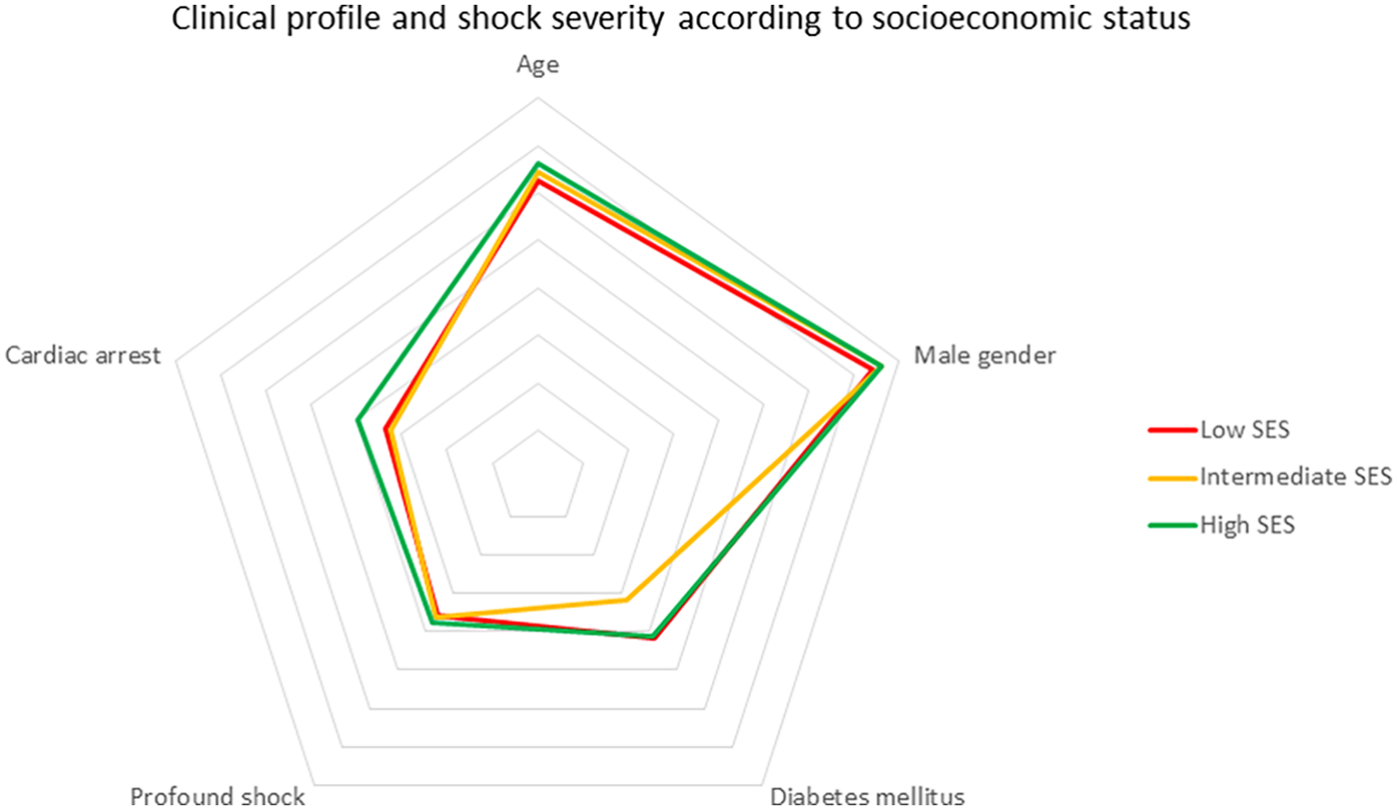
Clinical profile and shock severity according to socioeconomic status.

No significant differences were observed regarding age or gender across SES groups. The proportion of major comorbidities was similar across the different SES groups (Figure 1). The cause of CS was ACS more frequently in patients with low SES as compared to the rest (66.9% vs. 58%, *p* = 0.022). The severity of CS was similar across SES groups. While systolic blood pressure was slightly lower among patients from the high SES group, no significant differences were observed in other markers of CS severity such as cardiac arrest, renal function, lactate levels, left ventricular ejection fraction, or Cardshock score values (Table 1).

**Table 1:** Baseline clinical characteristics based on socioeconomic status (SES).

Clinical Characteristics	Low SES (*n* =125)	Intermediate SES (*n*=130)	High SES (*n*=127)	*p*-value
Age (years, mean, SD)	64.4 (16.0)	64.7 (13.0)	66.7 (13.0)	0.277
Male sex (*n*, %)	96 (74.2)	99 (76.0)	92 (76.0)	0.915
Arterial hypertension (*n*, %)	79 (63.7)	77 (59.2)	85 (67.5)	0.535
Dyslipidemia (*n*, %)	80 (64.5)	65 (50.0)	79 (62.7)	0.780
Active smoking (*n*, %)	39 (31.2)	48 (36.9)	34 (26.8)	0.448
Diabetes mellitus (*n*, %)	51 (41.1)	41 (31.8)	52 (41.3)	0.975
Peripheral artery disease (*n*, %)	14 (11.4)	16 (12.4)	16 (12.6)	0.770
Previous stroke (*n*, %)	16 (12.9)	7 (5.4)	14 (11.0)	0.626
Chronic obstructive pulmonary disease (COPD) (*n*, %)	18 (14.5)	15 (11.5)	16 (12.8)	0.688
Chronic kidney disease (*n*, %)	14 (11.5)	17 (13.1)	15 (11.8)	0.939
Previous neoplasia (*n*, %)	10 (8.1)	15 (11.5)	15 (11.9)	0.335
Cause of Shock				0.022
Acute coronary syndrome (*n*, %)	83 (66.9)	77 (59.2)	72 (56.7)	
Decompensated chronic heart failure (*n*, %)	20 (16.0)	19 (14.6)	15 (11.8)	
Electrical storm (*n*, %)	8 (6.5)	9 (6.9)	16 (12.6)	
Valvular heart disease (*n*, %)	1 (0.8)	8 (6.2)	3 (2.4)	
Myocarditis (*n*, %)	4 (3.2)	4 (3.1)	3 (2.4)	
Others (*n*, %)	8 (6.5)	13 (10.0)	18 (14.2)	
SCAI Shock Classification at Admission				0.743
A (*n*, %)	0	1 (0.8)	0	
B (*n*, %)	20 (16.0)	15 (11.5)	18 (14.2)	
C (*n*, %)	60 (48.0)	67 (51.5)	62 (48.8)	
D (*n*, %)	23 (18.4)	25 (19.2)	33 (26.0)	
E (*n*, %)	22 (17.6)	22 (16.9)	14 (11.0)	
Cardiac arrest (*n*, %)	41 (33.1)	42 (32.6)	50 (39.7)	0.272
Systolic blood pressure (mmHg)	92 (23)	95 (30)	85 (24)	0.011
Heart rate (bpm)	93 (29)	95 (29)	93 (34)	0.822
Glomerular filtration rate (mL/min/1.73 m²)	64 (34)	63 (29)	54 (25)	0.231
Cardshock score	4.2 (2)	4.1 (2)	4.6 (2)	0.106
Left ventricular ejection fraction (%)	31 (13)	33 (15)	32 (13)	0.489
Glucose at admission (mg/dL)	199 (82)	201 (106)	231 (166)	0.096
Hemoglobin (g/dL)	12.5 (2)	12.7 (2)	13.2 (2)	0.039
Lactate at admission (mmol/L)	5.9 (5)	5.5 (5)	5.5 (5)	0.789

### Management and outcomes according to SES subgroups

**Figure 2:**
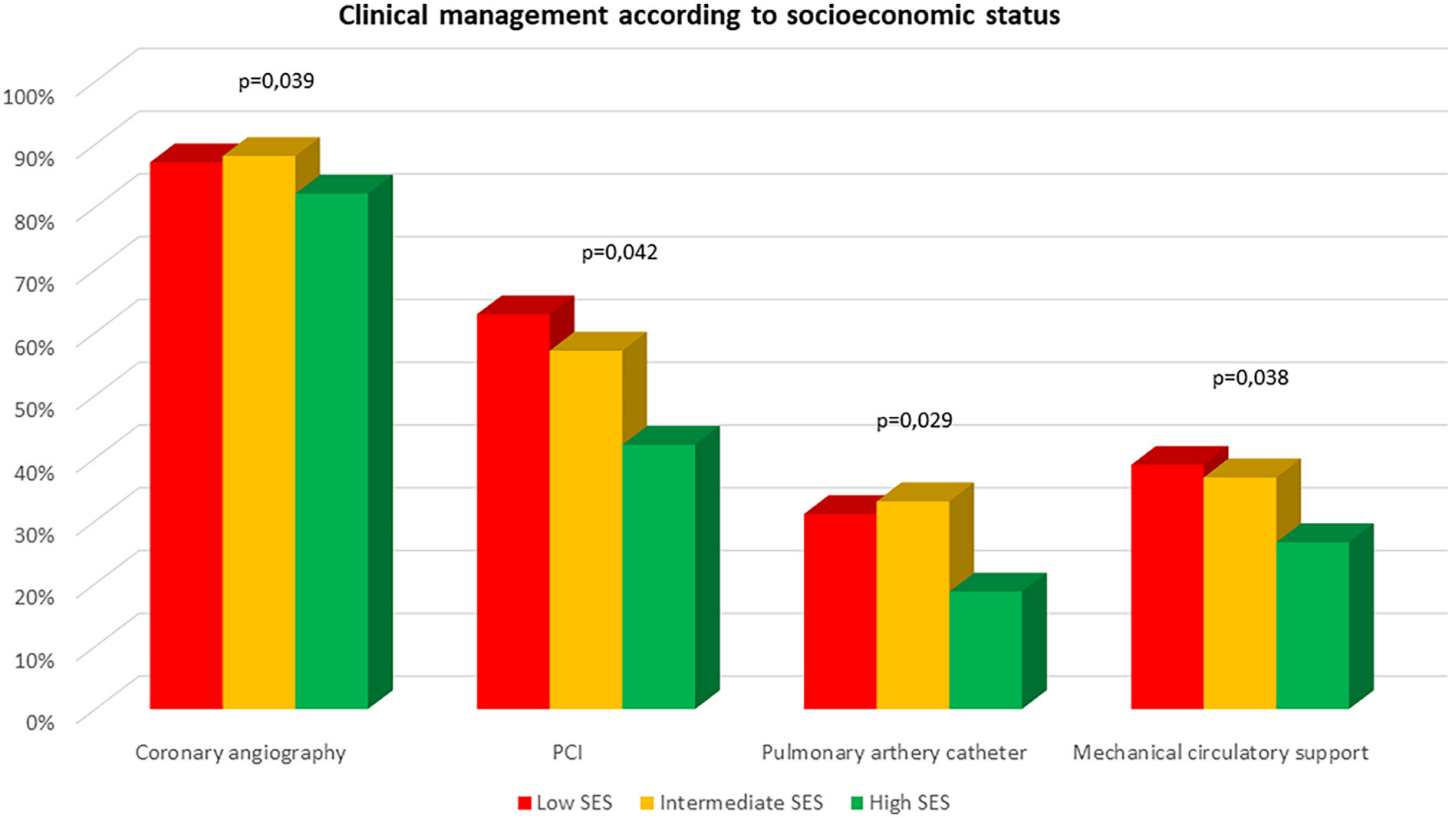
Clinical management according to socioeconomic status.

The crude analysis showed significant differences in the therapeutic approach among the different SES groups (Figure 2). Patients with low and intermediate SES underwent pulmonary artery catheterization, percutaneous coronary intervention (PCI), and MCS more frequently during hospitalization compared to the high SES group (Table 2). However, after adjusting for potential confounders, only a slight (non-significant) higher proportion of pulmonary artery catheter use was observed among patients from the low and intermediate SES groups (Supplementary Table S1c). No differences were observed regarding the rest of in-hospital procedures according to SES (Supplementary Tables S1a–f).

**Table 2:** Therapeutic approach and in-hospital outcomes according to socioeconomic status (SES).

Therapeutic approach	Low SES (*n* = 125)	Intermediate SES (*n* = 130)	High SES (*n* = 127)	*p*-value
Pulmonary artery catheter (*n*, %)	38 (31.1)	42 (33.1)	23 (18.7)	0.029
Coronary angiography (*n*, %)	108 (86.4)	111 (85.4)	101 (79.5)	0.139
Percutaneous coronary intervention (*n*, %)	78 (62.4)	72 (55.4)	60 (47.2)	0.016
Invasive mechanical ventilation (*n*, %)	78 (62.4)	82 (63.1)	81 (63.8)	0.772
Mechanical circulatory support (*n*, %)	49 (39.2)	48 (36.9)	34 (26.8)	0.038
Advanced circulatory support (*n*, %)	22 (17.6)	18 (13.8)	14 (11.0)	0.135
Extracorporeal renal replacement therapy (*n*, %)	21 (16.9)	10 (7.9)	20 (16.3)	0.873
Therapeutic hypothermia (*n*, %)	24 (19.4)	10 (15.5)	30 (24.2)	0.338
In-hospital Evolution				
Bleeding events (*n*, %)	36 (28.8)	29 (22.3)	20 (15.7)	0.018
Infectious complications (*n*, %)	67 (53.6)	58 (44.6)	69 (54.3)	0.647
Delirium (*n*, %)	27 (21.6)	19 (14.6)	22 (17.3)	0.379
In-hospital mortality (*n*, %)	49 (39.2)	34 (26.2)	40 (31.5)	0.194
ICU stay (mean, SD)	14 (13.0)	13 (12.0)	15 (15.0)	0.369
Hospital length of stay (mean, SD)	22 (21.0)	22 (22.0)	23 (22.0)	0.687

Patients in the lower SES groups experienced bleeding events more frequently during hospitalization (Table 2). No differences were observed in the incidence of delirium or infectious complications. Hospital length of stay and ICCU length of stay were similar across the three SES groups. Although mortality was slightly higher in the low SES group, this difference did not reach statistical significance. After adjusting for potential confounders, no association was observed between SES and mortality (Supplementary Table S2). Independent predictors of mortality included age, chronic kidney disease, severity of CS as measured by the SCAI class definition and cardiac arrest.

### Impact of socioeconomic status on therapeutic management and prognosis by sex and age

Supplementary Table S3 shows clinical profile, management and outcomes according to gender. Overall, women exhibited lower utilization rates of pulmonary artery catheter (17.9% vs. 29.3%, *p* = 0.029) and aMCS (6.3% vs. 16.4%, *p* = 0.015).

The impact of SES on clinical management varied slightly by gender. Among men, a higher proportion of patients in the low and intermediate SES groups received pulmonary artery catheterization (Supplementary Table S4). Additionally, a slightly higher use of PCI and MCS was observed in these patients, although this difference was statistically non-significant. Among women, the only significant difference was a higher utilization of aMCS in those from low and intermediate SES groups, with no significant differences in pulmonary artery catheter use or other invasive procedures (Supplementary Table S5). Regarding clinical outcomes, men from low and intermediate SES groups exhibited a higher incidence of bleeding complications, along with slightly increased mortality in these groups, without being statistically significant. In women, the only significant difference was a higher incidence of infectious complications in the high SES group. Hospital and ICCU length of stay did not significantly differ by SES in either men or women.

In general, older patients received pulmonary artery catheterization (19.3% vs. 37.4%, *p* < 0.001), MCS (28.7% vs. 40.8%, *p* = 0.013), and aMCS (6.9% vs. 22.3%, *p* < 0.001) less frequently. Once again, the impact of SES on treatment and prognosis was slightly different depending on age. Among younger patients, a slight non-significant higher use of pulmonary artery catheterization and MCS was observed in the low and intermediate SES groups (Supplementary Table S6). In contrast, among older patients, only a higher indication for PCI was noted in the low SES group (Supplementary Table S4). Younger patients from lower SES groups exhibited a significantly higher incidence of bleeding complications. However, these differences were not statistically significant in older patients. The incidence of other complications and in-hospital mortality was similar across SES groups both in younger and older patients. Finally, both ICCU and overall hospital length of stay were comparable across SES groups in younger patients. In contrast, among older patients, those in the higher SES group had a slightly shorter length of stay (both in the ICCU and overall hospitalization), although this difference did not reach statistical significance (Supplementary Table S7).

## Discussion

The main findings of this study are: (a) Patients with low SES admitted for CS in ICCU exhibited a similar clinical profile, comorbidity burden, and CS severity compared to other SES groups; (b) CS was more frequently caused by ACS in patients with low SES; (c) Clinical management adhered to guideline recommendations across SES groups; (d) Despite a higher incidence of bleeding in low SES patients, overall complication rates, in-hospital mortality, and length of stay were similar across SES groups.

Low SES has been associated with a higher comorbidity burden, lower adherence to guideline-directed therapy, and increased adverse event rates and mortality in various cardiovascular conditions, including chronic heart failure (6, 18), aortic stenosis (5), resuscitated sudden cardiac arrest (7), and coronary artery disease (4, 19). In ST-segment elevation myocardial infarction (STEMI), low SES is linked to worse clinical profiles and prolonged reperfusion times (20).

Data on the impact of SES on CS are scarce. Vallabhajosyula et al. (8) analyzed 402,182 patients with ACS-related CS in the United States (2000–2016), comparing uninsured patients to those with private insurance. Uninsured patients were younger, predominantly male, had lower SES, fewer comorbidities, and a higher incidence of multiorgan failure. Moreover, they were less likely to undergo coronary angiography, PCI, or MCS and had higher in-hospital mortality. Bloom et al. (9) examined 2,628 patients with CS transported by emergency medical services in Australia, reporting a progressive increase in CS incidence from the lowest to the highest SES quintile. Low SES patients were less likely to present to metropolitan hospitals and were more frequently treated at non-revascularization centers. These patients underwent fewer coronary angiographies and had higher 30-day mortality. The authors emphasized the need for equitable care in CS management regardless of SES. Similarly, Patlolla et al. (10) found that patients from lower-income areas (based on median district-level income) underwent fewer early coronary angiographies, PCI, MCS, and pulmonary artery catheterization and exhibited higher in-hospital mortality in a large cohort (*n* = 409,294) of patients with ACS-related CS. Other studies have also reported an association between low SES, reduced MCS utilization, and increased in-hospital mortality in this setting (11, 21). Notably, most data on the relationship between SES, treatment, and outcomes in CS derive from administrative databases, which often lack key clinical predictors. Furthermore, no data are available on the impact of SES on CS outcomes in Spain. This is a key aspect, since the public and universal nature of the healthcare system in this setting makes equity in the treatment of these highly complex patients a priority objective.

This is the first clinical registry to examine the association between SES, therapeutic approach, and prognosis in CS patients treated in routine clinical practice. The study design allowed for the analysis of key prognostic predictors (SCAI classification, lactate levels, heart rate, renal function), which are unavailable in most prior studies. In our cohort, SES was not associated with significant differences in clinical presentation or CS severity. Interestingly, a higher rate of ACS-related CS was observed among low SES patients, which may be closely related to the higher rate of PCI, other in-hospital procedures (MCS, pulmonary artery catheter) and in-hospital bleeding in this group. After adjusting for confounders, both management and outcomes were similar across SES subgroups These findings suggest a reasonably equitable approach to CS management in ICCU in our healthcare system, regardless of SES.

The analysis of the association between SES, therapeutic approach, and prognosis based on gender is particularly relevant, as significant interactions have been described between gender, the burden of cardiovascular risk factors, and patient outcomes (22). Data on the potential association between gender and SES in patients with CS are conflicting (8, 10). A different clinical profile and therapeutic approach have been described in women with CS, who tend to be older and present a higher burden of comorbidities. In a study by Sambola et al. (23), women with CS were less frequently referred to tertiary centers and had higher adjusted mortality compared to men. It has been suggested that women with acute heart failure often receive less optimized pharmacological treatment and fewer invasive procedures during hospitalization (24). Similarly, data from our series show that the use of pulmonary artery catheterization and aMCS was lower in women. Unlike other series, no differences in SES levels were observed between women and men in our study. On the other hand, some differences were noted in the impact of SES on the therapeutic approach based on gender, with greater use of aMCS in the lower SES groups among women. These findings should be interpreted with caution due to the size of the subgroups and require validation in larger cohorts. Given their potential relevance, the possible interactions between SES and gender in CS patients warrant further specific studies.

The interaction between SES and age is another important point, considering the ongoing population aging and the elevated risk faced by elderly patients. Older patients with CS receive MCS less frequently and are less likely to receive other recommended treatments, resulting in higher mortality (25). Additionally, a higher proportion of younger patients has been described in low SES groups (8, 10). Unlike these studies, data from our series did not show a significant association between age and SES. Older patients in this series received a more conservative approach, with less frequent use of pulmonary artery catheterization and MCS. The impact of SES on the therapeutic approach varied slightly by age, with a non-significant trend toward greater use of MCS in the low SES group only among younger patients. Again, the fragmentation of the sample limits the ability to draw robust conclusions from these data.

This study has several limitations. Its observational nature precludes the exclusion of residual confounding. Sample size was moderate, so these findings should be validated in larger cohorts. The inclusion of only ICCU-admitted patients may introduce selection bias, as the likelihood of ICCU admission may vary by SES (26). Data about excluded patients (postcardiotomy CS or those in whom CS was related to non-cardiac surgery) were not available. The SES index was assigned collectively to the entire population within each healthcare area, rather than on an individual basis, which may lead to an ecological bias. In addition, this SES score has not been previously validated in patients with CS. Therefore, this study was planned as a pilot study.

Despite these limitations, this study provides novel and relevant insights into the impact of SES on therapeutic strategies and prognosis in patients with CS managed in routine clinical practice within our healthcare system. In our view, the findings suggest a reasonably equitable approach to CS management, regardless of SES. Nevertheless, larger-scale studies are warranted to further optimize care strategies in this setting.

In conclusion, patients with low SES admitted to the ICCU for CS exhibit a similar clinical profile, comorbidity burden, and severity of CS compared to other patients. ACS was a more frequent etiology of CS in the low-SES group. Adherence to guideline-recommended therapy was observed across all SES groups. SES was not associated with significant differences in therapeutic management or prognosis in patients with CS admitted to the ICCU within routine clinical practice in our setting.

## Data Availability

The raw data supporting the conclusions of this article will be made available by the authors, without undue reservation.
