# Correction: Socioeconomic status and equity among patients with cardiogenic shock

**DOI:** 10.3389/fcvm.2025.1708500

**Published:** 2025-11-20

**Authors:** Marta Marcos-Mangas, Teresa López-Sobrino, Albert Ariza-Solé, Ferran Rueda-Sobella, Esther Sanz-Girgas, Jaime Aboal, Pablo Pastor, Irene Buera, Alessandro Sionis, Rut Andrea, Judit Rodríguez-López, Carlos Tomas, Jordi Bañeras, Isaac Llaó, José Carlos Sánchez-Salado, Cosme Garcia-Garcia

**Affiliations:** 1 Cardiology Department, Hospital Universitari Germans Trias I Pujol, Badalona, Spain; 2 Cardiology Department, Hospital Clínic de Barcelona Institut d’Investigacions Biomèdiques August Pi I Sunyer (IDIBAPS), Universitat de Barcelona, Barcelona, Spain; 3 Cardiology Department, Hospital Universitari Bellvitge, L’Hospitalet de Llobregat, Spain; 4 Cardiology Department, Hospital Universitari Joan XXIII, Tarragona, Spain; 5 Cardiology Department, Institut Investigació Sanitaria Pere Virgili, Tarragona, Spain; 6 Cardiology Department, Hospital Universitari Josep Trueta, Girona, Spain; 7 Cardiology Department, Hospital Arnau Vilanova, Lleida, Spain; 8 Cardiology Department, Hospital de la Vall d’ Hebrón, Barcelona, Spain; 9 Cardiology Department, Hospital Santa Creu I Sant Pau, II-B Sant Pau, Barcelona, Spain

**Keywords:** socioeconomic status, cardiogenic shock, intensive cardiac care units, mortality, intensive care management

The acknowledgements was erroneously omitted from the paper. The following acknowledgements has now been added “We thank CERCA Programme/Generalitat de Catalunya for institutional support.”

The original version of this article has been updated.

