# Supplementary material for: Socioeconomic status and equity among patients with cardiogenic shock
Source: Front Cardiovasc Med. 2025 Sep 9;12:1597225. doi: 10.3389/fcvm.2025.1597225 (PMC12454308; doi:10.3389/fcvm.2025.1597225)
Supplement: Supplementary file 1 [file Datasheet1.docx]

Supplementary material

*Supplementary tables. Multivariate analysis for predictors of in-hospital procedures*

*Supplementary table 1a. Multivariate analysis for predictors of angiography during the admission*

|  | *Univariate analysis* | | *Multivariate analysis* | |
| --- | --- | --- | --- | --- |
|  | *OR (95% CI)* | *P value* | *OR (95% CI)* | *P value* |
| *Age* | *1.00 (0.99-1.03)* | *0.475* | *1.03 (1.00-1.05)* | *0.025* |
| *Male gender* | *2.08 (1.13-3.85)* | *0.019* |  |  |
| *SES*  *High*  *Intermediate*  *Low* | *1*  *1.61 (0.79-3.28)*  *1.47 (0.73-2.96)* | *0.356* | *1*  *1.77 (0.78-3.99)*  *1.29 (0.57-2.89)* | *0.393* |
| *Center* | *1.04 (0.93-1.16)* | *0.479* |  |  |
| *Shock etiology* | *0.61 (0.53-0.72)* | *<0.001* | *0.589 (0.495-0.701)* | *<0.001* |
| *SCAI class* | *1.04 (0.75-1.43)* | *0.828* |  |  |
| *Cardiac arrest* | *2.03 (1.02.4.01)* | *0.043* | *2.07 (0.97-4.44)* | *0.062* |
| *Dyslipidemia* | *0.52 (0.28-0.99)* | *0.045* | *0.34 (0.15-0.76)* | *0.009* |
| *CKD* | *0.30 (0.15-0.62)* | *0.001* | *0.28 (0.12-0.66)* | *0.003* |
| *Prior neoplasm* | *0.43 (0.20-0.95)* | *0.037* |  |  |

*Supplementary table 1b. Multivariate analysis for predictors of percutaneous coronary intervention*

|  | *Univariate analysis* | | *Multivariate analysis* | |
| --- | --- | --- | --- | --- |
|  | *OR (95% CI)* | *P value* | *OR (95% CI)* | *P value* |
| *Age* | *1.01 (0.99-1.03)* | *0.177* |  |  |
| *Male gender* | *2.09 (1.29-3.37)* | *0.003* |  |  |
| *SES*  *High*  *Intermediate*  *Low* | *1*  *1.40 (0.85-2.31)*  *1.78 (1.07-2.96)* | *0.081* | *1*  *1.42 (0.75-2.67)*  *1.40 (0.75-2.62)* | *0.468* |
| *Center* | *1.07 (0.99-1.15)* | *0.082* | *1.08 (0.97-1.18)* | *0.160* |
| *Shock etiology* | *0.29 (0.0.21-0.39)* | *<0.001* | *0.29 (0.21-0.40)* | *<0.001* |
| *SCAI class* | *0.99 (0.79-1.25)* | *0.948* |  |  |
| *Cardiac arrest* | *1.21 (0.79-1.86)* | *0.390* |  |  |
| *Cardshock score* | *1.11 (0.98-1.25)* | *0.093* |  |  |
| *Lactate* | *0.96 (0.92-1.00)* | *0.056* |  |  |

*Supplementary table 1c. Multivariate analysis for predictors of pulmonary artery catheter insertion*

|  | *Univariate analysis* | | *Multivariate analysis* | |
| --- | --- | --- | --- | --- |
|  | *OR (95% CI)* | *P value* | *OR (95% CI)* | *P value* |
| *Age* | *0.96 (0.94-0.98)* | *<0.001* | *0.96 (0.94-0.98)* | *<0.001* |
| *Male gender* | *1.90 (1.06-3.42)* | *0.031* | *2.03 (1.05-3.93)* | *0.036* |
| *SES*  *High*  *Intermediate*  *Low* | *1*  *2.15 (1.19-3.86)*  *1.97 (1.09-3.56)* | *0.025* | *1*  *2.13 (1.12-4.05)*  *1.64 (0.84-3.18)* | *0.067* |
| *Center* | *1.00 (0.92-1.09)* | *9.938* |  |  |
| *Hypertension* | *0.54 (0.34-0.87)* | *0.010* |  |  |
| *CKD* | *0.52 (0.23-1.16)* | *0.111* |  |  |
| *Shock etiology* | *0.99 (0.86-1.13)* | *0.855* |  |  |
| *SCAI class* | *1.63 (1.26-2.10)* | *<0.001* | *1.69 (1.25-2.28)* | *0.001* |
| *Cardiac arrest* | *0.59 (0.36-0.98)* | *0.043* | *0.34 (0.18-0.63)* | *0.001* |
| *HR* | *1.01 (1.00-1.02)* | *0.002* |  |  |
| *LVEF* | *0.97 (0.95-0.98)* | *<0.001* | *0.98 (0.96-0.99)* | *0.019* |
| *Cardshock score* | *0.88 (0.77-1.01)* | *0.056* |  |  |
| *Lactate* | *1.04 (0.99-10.08)* | *0.096* |  |  |

*Supplementary table 1d. Multivariate analysis for predictors of mechanical circulatory support*

|  | *Univariate analysis* | | *Multivariate analysis* | |
| --- | --- | --- | --- | --- |
|  | *OR (95% CI)* | *P value* | *OR (95% CI)* | *P value* |
| *Age* | *0.98 (0.96-0.99)* | *0.025* |  |  |
| *Male gender* | *1.21 (0.73-2.01)* | *0.453* |  |  |
| *SES*  *High*  *Intermediate*  *Low* | *1*  *1.62 (0.95-2.75)*  *1.76 (1.03-2.99)* | *0.085* | *1*   - 1. *(0.74-2.68)*   *1.62 (0.85-3.09)* | *0.330* |
| *Center* | *1.03 (0.95-1.11)* | *0.440* | *1.01 (1.01-1.23)* | *0.028* |
| *Shock etiology* | *0.76 (0.65-0.89)* | *<0.001* | *0.69 (0.57-0.83)* | *<0.001* |
| *SCAI class* | *2.11 (1.64-2.71)* | *<0.001* | *3.62 (2.51-5.22)* | *<0.001* |
| *Cardiac arrest* | *0.49 (0.31-0.79)* | *0.003* | *0.21 (0.11-0.41)* | *<0.001* |
| *SBP* | *0.99 (0.98-1.01)* | *0.166* |  |  |
| *HR* | *1.01 (1.00-1.02)* | *0.008* |  |  |
| *LVEF* | *0.98 (0.96-0.99)* | *0.005* | *0.98 (0.97-1.00)* | *0.072* |
| *Cardshock score* | *1.01 (1.00-1.02)* | *0.008* |  |  |
| *Haemoglobin* | *0.99 (0.98-1.00)* | *0.155* |  |  |
| *Lactate* | *1.05 (1.01-1.10)* | *0.021* |  |  |

*Supplementary table 1e. Multivariate analysis for predictors of therapeutic hypothermia*

|  | *Univariate analysis* | | *Multivariate analysis* | |
| --- | --- | --- | --- | --- |
|  | *OR (95% CI)* | *P value* | *OR (95% CI)* | *P value* |
| *Age* | *1.01 (0.99-1.03)* | *0.475* | *1.03 (1.00-1.05)* | *0.030* |
| *Male gender* | *2.08 (1.13-3.85)* | *0.019* |  |  |
| *SES*  *High*  *Intermediate*  *Low* | *1*  *1.61 (0.79-3.28)*  *1.47 (0.73-2.96)* | *0.356* | *1*  *1.79 (0.79-4.02)*  *1.35 (0.60-3.01)* | *0.370* |
| *Center* | *1.04 (0.93-1.16)* | *0.479* |  |  |
| *Shock etiology* | *0.61 (0.53-0.72)* | *<0.001* | *0.59 (0.49-0.70)* | *<0.001* |
| *SCAI class* | *1.04 (0.75-1.43)* | *0.828* |  |  |
| *Cardiac arrest* | *2.03 (1.02-4.01)* | *0.043* | *2.15 (1.01-4.59)* | *0.049* |
| *Dyslipidemia* | *0.52 (0.28-0.99)* | *0.045* | *0.35 (0.16-0.78)* | *0.010* |
| *CKD* | *0.30 (0.15-0.62)* | *0.001* | *0.27 (0.12-0.63)* | *0.002* |
| *Prior neoplasm* | *0.43 (0.20-0.95)* | *0.037* |  |  |

*Supplementary table 1f. Multivariate analysis for predictors of invasive mechanical ventilation*

|  | *Univariate analysis* | | *Multivariate analysis* | |
| --- | --- | --- | --- | --- |
|  | *OR (95% CI)* | *P value* | *OR (95% CI)* | *P value* |
| *Age* | *0.97 (0.95-0.98)* | *<0.001* | *0.98 (0.96-0.99)* | *0.022* |
| *Male gender* | *1.82 (1.13-2.94)* | *0.014* |  |  |
| *SES*  *High*  *Intermediate*  *Low* | *1*  *0.93 (0.55-1.55)*  *0.90 (0.54-1.51)* | *0.919* | *1*   - 1. *(0.70-2.93)*   *0.96 (0.46-1.95)* | *0.445* |
| *Center* | *0.86 (0.80-0.93)* | *<0.001* | *0.91 (0.81-1.02)* | *0.093* |
| *Shock etiology* | *1.01 (0.88-1.14)* | *0.932* |  |  |
| *SCAI class* | *1.97 (1.51-2.58)* | *<0.001* | *1.82 (1.25-2.65)* | *0.002* |
| *Cardiac arrest* | *37.14 (13.3-103.7)* | *<0.001* | *41.5 (13.7-126.1)* | *<0.001* |
| *SBP* | *0.99 (0.98-0.99)* | *0.003* | *0.98 (0.97-0.99)* | *0.008* |
| *Hypertension* | *0.65 (0.42-1.03)* | *0.064* |  |  |
| *Prior stroke* | *0.50 (0.25-1.01)* | *0.053* |  |  |
| *CKD* | *0.63 (0.34-1.17)* | *0.140* |  |  |
| *Prior neoplasm* | *0.35 (0.18-0.69)* | *0.002* | *0.24 (0.09-0.67)* | *0.006* |
| *Cardshock score* | *0.99 (0.99-1.27)* | *0.062* |  |  |
| *Lactate* | *1.18 (1.09-1.26)* | *<0.001* | *1.10 (1.02-1.19)* | *0.016* |

*Supplementary table 2. Multivariate analysis for predictors of in-hospital mortalitu*

|  | *Univariate analysis* | | *Multivariate analysis* | |
| --- | --- | --- | --- | --- |
|  | *OR (95% CI)* | *P value* | *OR (95% CI)* | *P value* |
| *Age* | *1.03 (1.01-1.05)* | *0.001* | *1.05 (1.03-1.07)* | *<0.001* |
| *Male gender* | *0.89 (0.54-1.47)* | *0.654* |  |  |
| *SES*  *High*  *Intermediate*  *Low* | *1*  *0.79 (0.46-1.35)*  *1.37 (0.82-2.31)* | *0.118* | *1*  *0.68 (0.35-1.32)*  *1.69 (0.89-3.21)* | *0.197* |
| *Center* | *0.95 (0.88-1.03)* | *0.194* |  |  |
| *Shock etiology* | *0.98 (0.86-1.12)* | *0.773* |  |  |
| *SCAI class* | *3.04 (2.29-4.05)* | *<0.001* | *3.67 (2.63-5.12)* | *<0.001* |
| *Cardiac arrest* | *1.96 (1.25-3.06)* | *0.003* | *2.32 (1.32-4.11)* | *0.004* |
| *SBP* | *0.99-0.98-1.00)* | *0.019* |  |  |
| *Hypertension* | *1.64 (1.03-2.61)* | *0.038* |  |  |
| *CKD* | *4.52 (2.35-8.71)* | *<0.001* | *4.52 (2.01-10.2)* | *<0.001* |
| *Cardshock score* | *1.46 (1.27-1.69)* | *<0.001* |  |  |
| *Left ventricle ejection fraction* | *0.99 (0.97-1.01)* | *0.175* |  |  |
| *Lactate* | *1.08 (1.03-1.13)* | *0.001* |  |  |

Supplementary table 3. Clinical Characteristics, management and outcomes according to gender

|  | Males  (n=287) | Females  (n=95) | p-value |
| --- | --- | --- | --- |
| Age (years, median, P25-P75) | 64 (57-73) | 71 (63-81) | 0.002 |
| SES tertiles  Low  Intermediate  High | 93 (32.4)  99 (34.5)  95 (33.1) | 33 (34.7)  32 (33.7)  30 (31.6) | 0.930 |
| Arterial hypertension (n,%) | 175 (60.9) | 63 (66.3) | 0.323 |
| Active smoking (n,%) | 109 (37.9) | 11 (11.6) | <0.001 |
| Diabetes mellitus (n,%) | 108 (37.6) | 34 (35.8) | 0.764 |
| Peripheral artery disease (n,%) | 38 (13.2) | 6 (6.3) | 0.068 |
| Previous stroke (n,%) | 28 (9.8) | 8 (8.4) | 0.446 |
| Chronic kidney disease (n,%) | 38 (13.2) | 9 (9.5) | 0.248 |
| Cause of Shock |  |  | <0.001 |
| Acute coronary syndrome (n,%) | 192 (66.9) | 43 (45.3) |  |
| Decompensated chronic heart failure (n,%) | 41 (14.3) | 15 (15.8) |  |
| Electrical storm (n,%) | 23 (8) | 8 (8.4) |  |
| Valvular heart disease (n,%) | 7 (2.4) | 5 (5.3) |  |
| Myocarditis (n,%) | 5 (1.7) | 6 (6.3) |  |
| Others (n,%) | 19 (6.6) | 18 (19) |  |
| SCAI Shock Classification at Admission |  |  | 0.414 |
| A (n,%) | 0 | 1 (1.1) |  |
| B (n,%) | 41 (14.3) | 12 (12.6) |  |
| C (n,%) | 143 (49.8) | 45 (47.4) |  |
| D (n,%) | 60 (20.9) | 24 (25.3) |  |
| E (n,%) | 43 (15) | 13 (13.7) |  |
| Cardiac arrest (n, %) | 108 (37.6) | 23 (24.2) | 0.017 |
| Systolic blood pressure (mmHg) | 87 (76-100) | 85 (74-100) | 0.652 |
| Heart rate (bpm) | 96 (75-115) | 95 (70-114) | 0.220 |
| Left ventricular ejection fraction (%) | 30 (20-40) | 30 (22-40) | 0.299 |
| Lactate at admission (mmol/L) | 3.9 (2.4-7) | 3.5 (2.2-6.2) | 0.894 |
| Pulmonary artery catheter (n,%) | 84 (29.3) | 17 (17.9) | 0.029 |
| Coronary angiography (n,%) | 244 (85) | 71 (74.7) | 0.025 |
| Percutaneous coronary intervention (n,%) | 169 (69.3) | 39 (54.9) | 0.027 |
| Invasive mechanical ventilation (n,%) | 191 (66.6) | 49 (51.6) | 0.014 |
| Mechanical circulatory support (n,%) | 101 (35.2) | 29 (30.5) | 0.453 |
| Advanced circulatory support (n,%) | 47 (16.4) | 6 (6.3) | 0.015 |
| Renal replacement therapies (n,%) | 40 (13.9) | 11 (11.6) | 0.558 |
| Therapeutic hypothermia (n,%) | 62 (21.6) | 10 (10.5) | 0.018 |
| Bleeding events (n,%) | 66 (22.9) | 19 (20) | 0.522 |
| Infectious complications (n,%) | 156 (54.3) | 38 (40) | 0.011 |
| In-hospital mortality (n,%) | 90 (31.4) | 33 (34.7) | 0.654 |
| ICU stay (median, p25-75) | 8 (5-19) | 7 (3-16) | 0.628 |
| Hospital stay (median, p25-75) | 15 (8-27) | 13 (7-27) | 0.452 |

Supplementary table 4. Clinical management and in-hospital clinical course according to socioeconomic status among males.

|  | Low SES (n=93) | Intermediate SES (n=98) | High SES (n=96) | p-value |
| --- | --- | --- | --- | --- |
| Therapeutic Approach | | | | |
| Pulmonary artery catheter (n,%) | 31 (33.3) | 34 (34.7) | 19 (19.8) | 0.047 |
| Coronary angiography (n,%) | 82 (88.2) | 85 (86.7) | 77 (80.2) | 0.076 |
| Percutaneous coronary intervention (n,%) | 60 (64.5) | 58 (59.2) | 51 (53.1) | 0.090 |
| Invasive mechanical ventilation (n,%) | 64 (68.8) | 63 (64.3) | 62 (64.6) | 0.389 |
| Mechanical circulatory support (n,%) | 37 (39.8) | 37 (37.8) | 27 (28.1) | 0.081 |
| Advanced circulatory support (n,%) | 17 (18.3) | 16 (16.3) | 14 (14.6) | 0.471 |
| Extracorporeal blood purification (n,%) | 15 (16.1) | 8 (8.2) | 17 (17.7) | 0.673 |
| Therapeutic hypothermia (n,%) | 20 (21.5) | 18 (18.4) | 24 (25) | 0.502 |
| In-hospital Evolution | | | | |
| Hemorrhages (n,%) | 29 (31.2) | 22 (22.4) | 15 (15.6) | 0.007 |
| Infectious complications (n,%) | 57 (61.3) | 46 (46.9) | 52 (54.1) | 0.504 |
| Delirium (n,%) | 20 (21.5) | 12 (12.2) | 16 (16.7) | 0.155 |
| In-hospital mortality (n,%) | 36 (39.6) | 23 (24.7) | 30 (32.6) | 0.252 |
| ICCU stay (median, p25-75) | 8 (4-19) | 9 (5-17) | 8 (4-19) | 0.456 |
| Hospital stay (median, p25-75) | 15 (6-33) | 16 (10-26) | 13 (7-23) | 0.403 |

Supplementary Table 5. Clinical management and in-hospital clinical course according to socioeconomic status (SES) among females

| Variable | Low SES (n=32) | Intermediate SES (n=31) | High SES (n=32) | p-value |
| --- | --- | --- | --- | --- |
| Therapeutic Approach | | | | |
| Pulmonary artery catheterization (n,%) | 6 (18.8) | 7 (22.6) | 4 (12.5) | 0.510 |
| Coronary angiography (n,%) | 24 (75) | 25 (80.6) | 22 (68.8) | 0.888 |
| Percutaneous coronary intervention (n,%) | 17 (53.1) | 13 (41.9) | 9 (28.1) | 0.067 |
| Invasive mechanical ventilation (n,%) | 13 (40.6) | 18 (58.1) | 18 (56.2) | 0.332 |
| Mechanical circulatory support (n,%) | 11 (34.4) | 11 (35.5) | 7 (21.9) | 0.357 |
| Advanced circulatory support (n,%) | 4 (12.5) | 2 (6.5) | 0 | 0.046 |
| Renal replacement therapy (n,%) | 6 (18.8) | 2 (6.5) | 3 (9.4) | 0.258 |
| Therapeutic hypothermia (n,%) | 3 (9.4) | 2 (6.5) | 5 (15.6) | 0.388 |
| In-Hospital Evolution | | | | |
| Hemorrhages (n,%) | 7 (21.9) | 7 (22.6) | 5 (15.6) | 0.949 |
| Infectious complications (n,%) | 9 (28.1) | 12 (38.7) | 16 (50) | 0.041 |
| Delirium (n,%) | 6 (18.8) | 7 (22.6) | 5 (15.6) | 0.882 |
| In-hospital mortality (n,%) | 13 (40.6) | 10 (32.3) | 9 (28.1) | 0.378 |
| ICCU stay (median, p25-75) | 8 (4-18) | 7 (3-13) | 7 (3-16) | 0.809 |
| Hospital length of stay (median, p25-75) | 16 (8-28) | 12 (9-23) | 14 (4-27) | 0.590 |

Supplementary Table 6. Clinical management and in-hospital clinical course according to socioeconomic status (SES) among patients <65 years.

|  | Low SES (n=57) | Intermediate SES (n=69) | High SES (n=54) | p-value |
| --- | --- | --- | --- | --- |
| Therapeutic Approach | | | | |
| Pulmonary artery catheter (n, %) | 24 (42.9) | 28 (41.8) | 13 (25.5) | 0.069 |
| Coronary angiography (n, %) | 48 (84.2) | 60 (87) | 42 (79.2) | 0.494 |
| Percutaneous coronary intervention (n, %) | 31 (54.4) | 43 (62.3) | 26 (49.1) | 0.596 |
| Invasive mechanical ventilation (n, %) | 41 (71.9) | 45 (65.2) | 40 (75.5) | 0.345 |
| Mechanical circulatory support (n, %) | 27 (47.4) | 29 (42) | 17 (32.1) | 0.105 |
| Advanced circulatory support (n, %) | 15 (26.3) | 15 (21.7) | 10 (18.9) | 0.348 |
| Extracorporeal purification (n, %) | 8 (14) | 7 (10.3) | 9 (17.6) | 0.611 |
| Therapeutic hypothermia (n, %) | 13 (22.8) | 13 (18.8) | 16 (30.8) | 0.347 |
| In-Hospital Evolution | | | | |
| Hemorrhages (n,%) | 17 (29.8) | 15 (21.7) | 7 (13) | 0.049 |
| Infectious complications (n,%) | 33 (57.9) | 31 (44.9) | 33 (61.1) | 0.381 |
| Delirium (n,%) | 13 (22.8) | 6 (8.7) | 9 (16.7) | 0.377 |
| In-hospital mortality (n,%) | 17 (29.8) | 14 (20.3) | 11 (20.4) | 0.256 |
| ICCU stay (median, p25-75) | 8 (5-18) | 9 (5-18) | 10 (4-22) | 0.886 |
| Hospital length of stay (median, p25-75) | 12 (7-36) | 16 (10-25) | 15 (10-35) | 0.841 |

Supplementary Table 7. Clinical management and in-hospital clinical course according to socioeconomic status (SES) among patients ≥65 Years.

|  | Low SES (n=68) | Intermediate SES (n=60) | High SES (n=74) | p-value |
| --- | --- | --- | --- | --- |
| Therapeutic Approach | | | | |
| Pulmonary artery catheterization (n,%) | 14 (20.6) | 14 (23.3) | 10 (13.9) | 0.267 |
| Coronary angiography (n,%) | 60 (88.2) | 50 (83.3) | 59 (79.7) | 0.173 |
| Percutaneous coronary intervention (n,%) | 47 (69.1) | 28 (46.7) | 34 (45.9) | 0.006 |
| Invasive mechanical ventilation (n,%) | 37 (54.4) | 36 (60) | 41 (55.4) | 0.943 |
| Mechanical circulatory support (n,%) | 22 (32.4) | 19 (31.7) | 17 (23) | 0.213 |
| Advanced circulatory support (n,%) | 7 (10.3) | 3 (5) | 4 (5.4) | 0.260 |
| Renal replacement therapy (n,%) | 13 (19.4) | 3 (5.1) | 11 (15.3) | 0.508 |
| Therapeutic hypothermia (n,%) | 11 (16.4) | 6 (10.2) | 14 (19.4) | 0.606 |
| In-Hospital Evolution | | | | |
|  | Low SES (n=68) | Intermediate SES (n=60) | High SES (n=74) | p-value |
| Hemorrhages (n, %) | 19 (27.9) | 14 (23.3) | 13 (17.6) | 0.145 |
| Infectious complications (n, %) | 34 (50) | 27 (45) | 36 (48.6) | 0.936 |
| Delirium (n, %) | 14 (20.6) | 13 (21.7) | 13 (17.6) | 0.646 |
| In-hospital mortality (n, %) | 32 (47.1) | 19 (31.7) | 29 (39.2) | 0.357 |
| ICCU stay (median, p25-75) | 8 (4-20) | 8 (5-14) | 5.5 (3-14) | 0.109 |
| Hospital stay (median, p25-75) | 15 (6-27) | 14 (9-26) | 10 (4-22) | 0.063 |
